# Larval Mortality and Ovipositional Preference in *Aedes albopictus* (Diptera: Culicidae) Induced by the Entomopathogenic Fungus *Beauveria bassiana* (Hypocreales: Cordycipitaceae)

**DOI:** 10.1093/jme/tjac084

**Published:** 2022-07-07

**Authors:** John M Kirsch, Jia-Wei Tay

**Affiliations:** Urban Entomology Laboratory, Department of Plant and Environmental Protection Sciences, University of Hawaii at Manoa, Gilmore, Honolulu, USA; Urban Entomology Laboratory, Department of Plant and Environmental Protection Sciences, University of Hawaii at Manoa, Gilmore, Honolulu, USA

**Keywords:** entomopathogenic fungi, larvae bioassay, oviposition, ovitrap, biological control

## Abstract

Entomopathogenic fungi allow chemical-free and environmentally safe vector management. *Beauveria bassiana* (Balsamo-Crivelli) Vuillemin is a promising biological control agent and an important component of integrated vector management. We investigated the mortality of *Aedes albopictus* (Skuse) larvae exposed to five concentrations of *B. bassiana* using Mycotrol ESO and adult oviposition behavior to analyze the egg-laying preferences of wild *Ae. albopictus* in response to different fungal concentrations. We examined the mortality of mid-instars exposed to *B. bassiana* concentrations of 1 × 10^4^, 1 × 10^5^, 1 × 10^6^, 1 × 10^7^, and 1 × 10^8^ conidia/ml every 24 h for 12 d. In the oviposition behavior study, the fungus was applied to wooden paddles at 1 × 10^5^, 1 × 10^7^, and 1 × 10^9^ conidia/ml, and the paddles were individually placed into quad-ovitraps. Both experiments contained control groups without *B. bassiana*. Kaplan–Meier survival analysis revealed that larval mortality was concentration dependent. The median lethal concentration was 2.43 × 10^5^ conidia/ml on d 12. The median lethal time was 3.68 d at 1 × 10^6^ conidia/ml. Oviposition monitoring revealed no significant difference in egg count between the control and treatment paddles. We observed an inverse relationship between the concentration of *B. bassiana* and the percentage of paddles with eggs. We concluded that concentrations above 1 × 10^6^ conidia/ml are larvicidal, and *Ae. albopictus* laid similar numbers of eggs on fungus-impregnated and control wooden substrates; however, they were more likely to oviposit on substrates without *B. bassiana*. With these findings, we suggest that *B. bassiana-*infused ovitraps can be used for mosquito population monitoring while also delivering mycopesticides to adult mosquitoes.

The Asian tiger mosquito, *Aedes albopictus* (Skuse), is considered one of the “world’s worst invasive alien species” owing to its ability to transmit human and animal diseases and establish itself in a wide range of environments (Lowe et al. 2004). *Aedes albopictus* breeds in containers and cavities from anthropogenic and natural sources and is able to survive in a wide variety of urban, suburban, and forested habitats (Paupy et al. 2009, Wilson et al. 2020). One of the ultimate goals of vector control, as outlined by the World Health Organization (WHO), is reducing human-vector contact and vector survival (WHO 2017). Worldwide, current management tactics, such as chemical pesticide application, have failed to alleviate biting pressure while contributing to the development of insecticide resistance to multiple classes of insecticides, including pyrethroids and organophosphates (Moyes et al. 2017). Insecticide resistance necessitates the use of 21st century integrated vector management tactics, with special attention given to environmentally safe strategies.

The entomopathogenic fungus *Beauveria bassiana* (Balsamo-Crivelli) Vuillemin (CAS No. 63428-82-0) is a biological control agent that can be incorporated into integrated pest management strategies. Mycopesticides, in rotation with other management tactics, have great potential to slow insecticide resistance development through population suppression and mosquito longevity reduction (Achee et al. 2019). *Beauveria bassiana* infects hosts through contact with the cuticle, where it adheres and germinates; it then penetrates the insect and extracts hemolymph nutrients, eventually killing the host (Litwin et al. 2020). It has also been proposed that larval death occurs due to secondary metabolites produced by the fungus and mechanical blockage of the siphon and trachea (Clark et al. 1968, Daniel et al. 2016). Entomopathogenic fungi are currently utilized on a variety of hexapod families in agriculture and ornamental crops (Arthurs and Dara 2019, Mascarin et al. 2019), and *B. bassiana* has been approved for use in In2Care mosquito traps (In2Care BV, Wageningen, Netherlands, EPA Reg. No. 91720-1) (Snetselaar et al. 2014, Su et al. 2020).

The existing literature evaluating *B. bassiana* as a vector control agent generally focuses on either larvae or adults and either *Aedes aegypti* (Linnaeus) or *Anopheles gambiae* Giles (Diptera: Culicidae), with few studies analyzing *Ae. albopictus* in the two developmental stages (Deng et al. 2017, 2019a). Studies evaluating mosquito larval mortality after exposure to various strains of *B. bassiana* or secondary metabolites produced by the fungus aim to reduce the vector population before adulthood and therefore reduce the risk of disease transmission (Clark et al. 1968, Miranpuri and Khachatourians 1991, Montoya-Treviño et al. 2008, Alcalde-Mosqueira et al. 2014, Daniel et al. 2016, Deng et al. 2017, Vivekanandhan et al. 2018, Deng et al. 2019a, de Luna-Santillana et al. 2020, Vivekanandhan et al. 2020). Additionally, the mortality rates in adult *Aedes* exposed to *B. bassiana* varied between 60% and 100%, depending on the length of study and exposure amount, with most studies reporting 80–90% mortality rates (de Paula et al. 2008, Darbro et al. 2012, Deng et al. 2019b, Lee et al. 2019, de Luna-Santillana et al. 2020, Shoukat et al. 2020). However, the aforementioned studies did not analyze fungus-induced behavior and rather focused on olfactory and visual attractants. These strong stimuli likely conceal fungus-induced attraction or repulsion, resulting in unexplored areas involving fungus-induced behavior (Snetselaar et al. 2014, Buckner et al. 2017, Paula et al. 2018, Silva et al. 2018). When mycopesticides are used for the control of adult mosquitoes, they must be applied at a concentration that causes pathogenicity but does not deter contact between the pathogen and host.

In this study, a larval bioassay investigated the mortality of second- and third-instar *Ae. albopictus* exposed to five different concentrations of *B. bassiana* in Mycotrol ESO. The second part of the study investigated wild *Ae. albopictus* ovipositional behavior to determine if three different concentrations of *B. bassiana* altered the number of eggs laid and percent ovipositon in the ovitraps. With an understanding of these preferences, future vector management research can determine how the use of this entomopathogen would benefit surveillance and management programs.

## Materials and Methods

### Larval Bioassay

#### Mosquitoes

*Aedes albopictus* were collected using ovipositional traps from the University of Hawai‘i at Mānoa campus and maintained in a colony. Eggs were hatched in distilled water, and larvae were maintained at ambient temperature and humidity (22 ± 1.5°C, 65 ± 10%), with a 12L:12D photoperiod. Larvae were fed a 1:1:1 mixture of brewer’s yeast, skim milk powder, and bovine liver powder (MP Biomedicals LLC., Solon, OH, USA) with 0.4% Vanderzants Vitamin Mixture for Insects (MP Biomedicals LLC., Solon, OH, USA). Adults were provided 10% (w/v) sucrose *ad libitum*. Adult females were periodically provided a blood meal of defibrinated bovine blood (HemoStat Laboratories, Dixon, CA, USA) warmed at 40°C for a minimum of 20 min via a parafilm-wrapped Petri dish with a heat pack for warmth (Moutailler et al. 2007). Damp filter paper folded in a cone on a Petri dish was provided as an egg-laying substrate and replaced when needed.

#### Fungus Preparation

*Beauveria bassiana* strain GHA was purchased in the form of Mycotrol ESO (LAM International Corporation, Butte, Montana, USA). Stock Mycotrol ESO was halved with distilled water at a concentration of 1.06 × 10^10^ conidia/ml (rounded to 1 × 10^10^). From the stock solution, serial dilutions were prepared using distilled water. Five concentrations of *B. bassiana* were tested, in addition to a control without fungus. Furthermore, 0.05% of polysorbate 20 (Sigma–Aldrich, St. Louis, Missouri, USA) was added to each treatment and control to reduce formula separation (Dong et al. 2012). Experimental fungus concentrations were based on previous studies and WHO guidelines (WHO 2005; Deng et al. 2017, 2019a).

#### Bioassay

Twenty-five second- and third-instar *Ae. albopictus* larvae were added by a transfer pipette to containers with 100 ml of distilled water. Each serial dilution was dispensed into the larval containers for final concentrations of 1 × 10^8^, 1 × 10^7^, 1 × 10^6^, 1 × 10^5^, and 1 × 10^4^ conidia/ml of *B. bassiana* and a control. Each trial comprised four containers for each treatment and the control. Each container was provided food and maintained at ambient temperature and humidity (22 ± 1.5°C, 65 ± 10%). Mortality was monitored every 24 h for 12 d using established WHO guidelines (WHO 2005). Up to ten mosquito larvae from each container were separated upon death and placed individually in a Petri dish with dampened black filter paper. The dishes were placed in an incubator (18 ± 2°C, RH 65 ± 10%), and fungal presence was later confirmed on the larval carcasses. The experiment was repeated three times, with a total of 300 larvae tested in each treatment and control.

### Oviposition Behavior

#### Trap and Fungus Preparations

Mycotrol ESO was diluted with distilled water to produce concentrations of 1 × 10^5^, 1 × 10^7^, and 1 × 10^9^ conidia/ml. The diluted concentrations of *B. bassiana* were applied to all sides of a wooden paddle with a paint brush and allowed to dry for three h. Twenty-four 473-ml (16 oz.) black plastic cups with five small holes placed at two-thirds the height (approx. 300 ml) were arranged in groups of four to create quad-ovitraps. Three wooden paddles (2.5 × 14.0 × 0.5 cm) on which each concentration of *B. bassiana* (treatment) was applied and one paddle on which no *B. bassiana* applied (control) were set in individual cups of the quad-ovitrap, for a total of four paddles (Supp. Fig. 1 [Online only]). Each cup was filled with tap water to the overflow holes. Ovitraps were placed in the environment for seven days before servicing, which included collecting and replacing the wooden paddle and tap water. Trapping was conducted for six weeks, totaling 128 trapping events.

#### Location

The study was conducted at six sites, with one quad-ovitrap placed at each site. The sites were located on the University of Hawai‘i at Mānoa campus (21.301° N, 157.816° W) at approximately 30 m elevation (USGS 2021). The study was conducted during the end of the transition from the wet winter season to the drier summer season from February-May 2021. The average temperature during the study was 23.44°C, and the average relative humidity was 69.45%. The average weekly rainfall was 2.42 mm. Climatology data were obtained from the Department of Atmospheric Sciences at the University of Hawai‘i at Mānoa (UH Mānoa Atmospheric Sciences 2021).

#### Sample Processing

The collected paddles were placed on paper towels for two days to dry. Eggs were counted using a dissecting microscope (Leica M80, Leica Instruments Pte Ltd., Germany). Paddles without eggs were placed in an incubator to allow fungal growth to determine viability. After a two-day embryonation period, paddles with eggs were submerged into individual containers with distilled water and 0.05 g of yeast. Paddles were left submerged for one week to allow hatching. Hatched larvae were reared to adulthood, at which point they were frozen and identified to species (Darsie and Ward 2016).

### Statistical Analysis

For the larval bioassay, a Kaplan–Meier survival analysis was performed using the R package ‘survival’ v.3.2-7, followed by a log-rank test with pairwise comparisons to determine differences in mosquito survivorship between the treatments and the control (Therneau 2021). Lethal concentrations (LC_50_, LC_90_, LC_99_) at d 12 and median lethal time (LT_50_) were calculated using a probit analysis in the R package ‘ecotox’ v.1.4.2 (Hlina 2021).

For the oviposition behavior experiment, a generalized linear model (GLM) with negative binomial distribution was constructed to determine if there was a difference in the number of eggs laid among the three concentrations of fungus and the control. The goal of the model was to evaluate if concentration of fungus and site had influences on the number of eggs laid on the paddles. The model was constructed using R package ‘MASS’ and best fit was selected using AICc in R package ‘bbmle’ (Akaike 1973, Venables and Ripley 2002, Bolker and R Development Core Team 2020).

Subsequently, a presence or absence GLM with a binomial distribution was constructed to determine if there was a difference in the percentage of paddles with and without eggs by concentration. All paddles that had one or more egg were included in the “present” category, while all paddles that had zero eggs were included in the “absent” category. A log-likelihood ratio test was performed against a null model to determine if the concentration significantly influenced the presence of mosquito eggs. All analyses were performed using R software v. 1.0.143 (R Core Team 2020).

## Results

### Larval Bioassay

#### Kaplan–Meier Survival Analysis

**Fig. 1.**
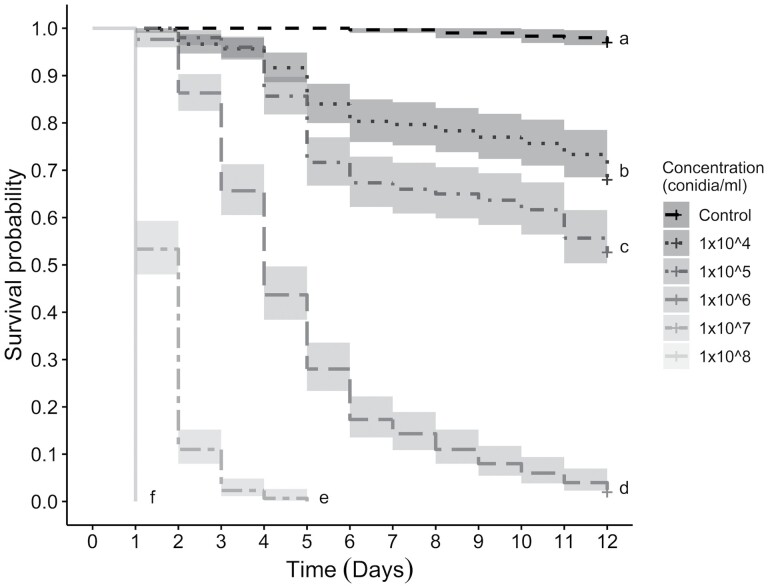
Kaplan–Meier survival curve of *Ae. albopictus* (*n* = 300) exposed to different concentrations of *B. bassiana*. All curves show significantly altered survival probabilities due to exposure to different fungus concentrations (χ^2^ = 67.50, df = 3, *P* < 0.0001). Letters following the survival curves show significant pairwise comparison values (all *P* < 0.0001). Censored data are shown as crosses at the end of the curves.

We conducted a Kaplan–Meier survival analysis followed by pairwise log-rank tests to determine the significant differences among each tested concentration. There was an overall significant effect of fungus concentration on survival of the mosquitoes (χ^2^ = 67.50, df = 3, p < 0.0001), with all pairwise comparisons resulting in significance; therefore, each concentration of fungus resulted in a different rate of mortality (Fig. 1).

#### Lethal Concentration

On d 12, the percent mortality for the 1 × 10^8^ and 1 × 10^7^ conidia/ml groups was 100% and for the 1 × 10^6^ conidia/ml concentration group, it was slightly lower, at 98.00 ± 0.60%. In the 1 × 10^5^ conidia/ml group, the mortality was 47.33 ± 8.55%, and for the 1 × 10^4^ conidia/ml group, the mortality was 32.00 ± 7.24%. In the control group, the percent mortality was 3.00 ± 1.00%. According to the probit analysis, the LC_50_, LC_90_, and LC_99_ values at d 12 were 2.43 × 10^5^ (2.07 × 10^5^–2.79 × 10^5^), 6.65 × 10^5^ (5.79 × 10^5^–7.52 × 10^5^), and 1.01 × 10^6^ (8.76 × 10^5^–1.14 × 10^6^) conidia/ml, respectively (Table 1).

**Table 1.** Percent mortality of *Ae. albopictus* larvae exposed to different concentrations of *B. bassiana* at d 12 (*n* = 300)

Concentration (conidia/ml)	Percent mortality (mean ± SE)	LC_50_ (LCL–UCL)	LC_90_ (LCL–UCL)	LC_99_ (LCL–UCL)	Slope ± SE	χ^2^	df	*P*-value
1 × 10^8^	100.00 ± 0.00	2.43 × 10^5^ (2.07 × 10^5^ – 2.79 × 10^5^)	6.65 × 10^5^ (5.79 × 10^5^ – 7.52 × 10^5^)	1.01 × 10^6^ (8.76 × 10^5^ – 1.14 × 10^6^)	1.08 ± 0.06	67.50	3	<0.0001
1 × 10^7^	100.00 ± 0.00							
1 × 10^6^	98.00 ± 0.60							
1 × 10^5^	47.33 ± 8.55							
1 × 10^4^	32.00 ± 7.24							
Control	3.00 ± 1.00							

SE = standard error, LC = lethal concentration (conidia/ml), LCL = 95% lower confidence level, UCL = 95% upper confidence level, χ^2^ = chi-square statistic, df = degrees of freedom.

#### Lethal Time

For each tested concentration, the LT_50_ was calculated using probit analysis. At 1 × 10^8^ conidia/ml, all larvae died before the first mortality check; therefore, no accurate measure could be calculated. At 1 × 10^7^ conidia/ml, the LT_50_ was 1.05 (0.97–1.12) d. At 1 × 10^6^ conidia/ml, the LT_50_ was 3.68 (3.54–3.81) d. At 1 × 10^5^ conidia/ml, the LT_50_ was 11.85 (10.23–14.60) d, which was slightly longer than the length of time *Ae. albopictus* spend as larvae before pupation in a favorable environment. At 1 × 10^4^ conidia/ml, the LT_50_ was 22.43 (19.32–27.14) d, exceeding the standard developmental time.

### Oviposition Behavior

#### Egg Count by Concentration

From 128 trapping events over six weeks, 1966 eggs were collected. The highest concentration of *B. bassiana*, 1 × 10^9^ conidia/ml, resulted in the fewest total eggs (216 eggs (6.75 ± 2.78 eggs/paddle)). The concentration with the most eggs was 1 × 10^7^ conidia/ml, with 627 eggs (19.59 ± 7.27 eggs/paddle). The lowest concentration, 1 × 10^5^ conidia/ml, resulted in a total of 556 eggs (17.38 ± 5.51 eggs/paddle), and the control resulted in 567 eggs (17.72 ± 5.61 eggs/paddle). All reared mosquitoes were identified as *Ae. albopictus*.

The best performing negative binomial distribution GLM for the egg count predictions contained the factors of concentration of fungus and site (Table 2). There was a statistically significant decrease in the amount of eggs laid with increasing fungus concentrations (95% CI: (−2.03 × 10^−9^–−2.13 × 10^−10^), *P* = 0.0104). The negative association indicated there was a deterring effect caused by the fungus to the mosquito when a gravid mosquito is scouting for oviposition locations. A likelihood ratio test between the best model and a null model resulted in significance demonstrating the concentration of fungus improved the model performance (χ^2^ = 5.403, df = 1, *P* = 0.0201). Significant site variations in the number of eggs were also determined by the model.

**Table 2.** Best performing generalized linear models for ovipositional preferences

Independent variable	Effects	Distribution	Factor	Estimate ± SE	Confidence interval	*P*-value
Number of eggs	Concentration + Site	Negative binomial	Intercept	0.27 ± 0.48	−0.58, 1.31	0.5760
			Concentration	−1.17 × 10^−9^ ± 4.57 × 10^−10^	−2.03 × 10^−9^, −2.13 × 10^−10^	0.0104*
			Site 2	2.94 ± 0.64	1.66, 4.21	<0.0001***
			Site 3	2.12 ± 0.64	0.84, 3.41	0.0009***
			Site 4	2.87 ± 0.64	1.59, 4.15	<0.0001***
			Site 5	3.47 ± 0.64	2.20, 4.76	<0.0001***
			Site 6	−2.19 ± 1.36	−5.40, 0.41	0.1078
Presence and absence of eggs	Concentration + Site	Binomial	Intercept	−1.10 ± 0.51	−2.22, −0.16	0.0318*
			Concentration	−1.23 × 10^−9^ ± 4.73 × 10^−10^	−2.20 × 10^−9^, −3.30 × 10^−10^	0.0092**
			Site 2	1.58 ± 0.67	0.32, 2.96	0.0176*
			Site 3	1.76 ± 0.67	0.50, 3.15	0.0085**
			Site 4	1.58 ± 0.67	0.32, 2.96	0.0176*
			Site 5	2.58 ± 0.71	1.25, 4.08	0.0003***
			Site 6	−0.63 ± 1.19	−3.68, 1.46	0.6002

SE = standard error, 95% confidence interval, best performing models ranked by AICc, significance symbols: 0.05 to 0.01 = *, 0.01 to 0.001 = **, <0.001 = ***.

#### Presence/Absence of Eggs by Concentration

The lowest percentage of paddles with eggs, 31.25% (10 out of 32) was associated with the highest concentration of fungus, 1 × 10^9^ conidia/ml. At 1 × 10^7^ conidia/ml, 50% (16 out of 32) of the paddles contained eggs. The lowest concentration, 1 × 10^5^ conidia/ml, had the highest percentage of paddles with eggs among the three treatments, with 56.25% (18 out of 32) of paddles containing eggs. The control had the highest percentage of paddles with eggs, at 62.5% (20 out of 32).

The GLM model created to assess the effect of concentration of fungus and site location on the presence and absence of mosquito eggs determined the best fit model contained the fixed effects of concentration and site (Table 2). Results showed there was a significant decline in the likelihood of eggs with an increase in fungus concentrations (95% CI: (−2.20 × 10^−9^–−3.30 × 10^−10^), *P* = 0.0092). The significant negative association between occurrence and concentration indicates selective behavior when determining an oviposition location. A likelihood ratio test resulted in a significant improvement with addition of fungus concentration (χ^2^ = 7.247, df = 1, *P* = 0.0071). Additionally, there were significant differences in egg presence between sites in the model.

## Discussion

Larval survival was significantly altered by the concentration of *B. bassiana*. The LC_50_ values for second- and third-instar *Ae. albopictus* in the current study were similar to the median lethal concentrations calculated by de Luna-Santillana et al. (2020) at 7–8 d with 10^5^–10^6^ conidia/ml. The LC_50_ values calculated by Alcalde-Mosqueira et al. (2014) and Montoya-Treviño et al. (2008) were both higher than the current findings LC_50_ of 2.43 × 10^5^ conidia/ml, as they reported LC_50_ values of 3.6 × 10^6^ conidia/ml and 3.86 × 10^6^ conidia/ml, respectively. These three previous studies examined the lethal concentrations for various strains of *B. bassiana* against third- and fourth-instar *Ae. aegypti*. The age of larvae at introduction of the fungus has been observed to determine susceptibility, with faster-growing species experiencing lower mortality attributable to fungal exposure (Clark et al. 1968, Scholte et al. 2004). The current experiment used both second- and third-instar larvae hatched at the same time, and the second instars may have survived exposure to a higher concentration due to molting soon after exposure to the fungus. Several environmentally sampled strains of *B. bassiana* have been shown to require higher concentrations of conidia to cause equivalent pathogenicity (Vivekanandhan et al. 2018, de Luna-Santillana et al. 2020). For example, *B. bassiana* sampled from southern Indian soils was used in a larval bioassay involving *Ae. aegypti*, and the results indicated an LT_50_ of 5.91 d and 78.66% mortality after 10 d of exposure to 1 × 10^8^ conidia/ml (Vivekanandhan et al. 2020). These findings at 1 × 10^8^ conidia/ml indicate lower pathogenicity than that in our experiment, in which all larvae died within 24 h.

A larval mortality trend was observed: there were two to three days with a high mortality rate, with the onset being staggered by concentration, followed by gradual mortality resulting in similar daily mortality percentages. This was observed for all three concentrations, 1 × 10^4^, 1 × 10^5^, and 1 × 10^6^ conidia/ml, from d 7 to study conclusion (Fig. 1), diverging from the control group. There were no consecutive days in which the control exhibited a similar mortality range. The continued mortality after the large initial mortality event suggests lingering fungal effects on the larvae, potentially weakening the immune system and other vital pathways (Yassine et al. 2012, Ramirez et al. 2019, Shoukat et al. 2020).

The question of whether *B. bassiana*, secondary metabolites, or other factors were the cause of the rapid mortality observed in our experiment remains to be elucidated. Because Mycotrol ESO contains petroleum distillates, it is possible that the larval environment became coated in an impermeable layer of oils, causing the larvae to die due to suffocation as opposed to fungal exposure, although the addition of polysorbate 20 reduced this possibility. By subsampling dead larvae, fungal growth was visually confirmed; however, the rapid mortality observed at the two highest concentrations does not accommodate the generalized timeline of the fungus (Clark et al. 1968). Secondary metabolites have been isolated and shown to cause larval mortality in past experiments (Daniel et al. 2016, Vivekanandhan et al. 2018, Zhang et al. 2020). Both our research team and previous researchers observed melanization of larval siphons and midguts during experimental periods, which is part of the immune response to fungal infection (Clark et al. 1968, Yassine et al. 2012). Mechanical blockage of the tracheal trunks and larval siphon by the fungus has been suggested as one of the causes of larval mortality (Clark et al. 1968, Miranpuri and Khachatourians 1991, Daniel et al. 2016, Amobonye et al. 2020). We hypothesize that multiple insecticidal modes of action occurred, causing initial and sustained mortality.

Genetic engineering of *B. bassiana* has been proposed to increase the efficacy of the fungus in second instar *Ae. albopictus* through increased and quicker lethality (Deng et al. 2017, 2019a). These studies hypothesized that the integration of a single-chain neurotoxic polypeptide (AaIT) from the venom of the buthid scorpion (*Androctonus australis*) or a toxin expressed by *Bacillus thuringiensis* (Cyt2Ba) into the genome of *B. bassiana* will cause increased virulence and decrease the survival of *Ae. albopictus* (Deng et al. 2017, 2019a). The current experiment with *B. bassiana* strain GHA resulted in an LC_50_ of 2.43 × 10^5^ conidia/ml at d 12, whereas the LC_50_ of the toxin-infused *B. bassiana* strain GIM3.428 was 1.47 × 10^3^ conidia/ml at d 10, and the LC_50_ of a wild type of the same strain was 1.65 × 10^4^ conidia/ml at d 10 (Deng et al. 2017). It would appear that the toxin-infused strain caused mortality at a lower concentration than the commonly used entomopathogenic strains. However, the *B. bassiana* strain GHA had a shorter LT_50_ of 1.05 d at 1 × 10^7^ conidia/ml and 3.68 d at 1 × 10^6^ conidia/ml compared to the Aa-IT toxin-infused strain, with an LT_50_ at 4.4 d and 5.5 d, respectively, and the Cyt2Ba toxin-infused strain, with an LT_50_ of 4.0 d and 5.5 d, respectively (Deng et al. 2017, 2019a). This can likely be attributed to Mycotrol ESO formula optimization as a pesticide.

When assessing whether adult behavior was altered by different concentrations of *B. bassiana*, we found that control paddles and paddles coated with 1 × 10^5^ conidia/ml more commonly had eggs than paddles coated with 1 × 10^7^ and 1 × 10^9^ conidia/ml. Model results show a significant negative association between the presence of mosquito eggs and the concentration of *B. bassiana*. The declining percentage of paddles with eggs with increasing concentration may be explained by the fungus itself or the product formulation. The fungus-impregnated wooden substrate may have been a deterrent to gravid mosquitoes, as hyphae or product oils potentially made the paddle unfavorable for oviposition. Additionally, secondary metabolites produced by the fungus may cause a repulsive effect in *Ae. albopictus*. However, little is known about secondary metabolite interactions with mosquitoes, and a previous study speculated that secondary metabolites have minimal behavioral influences on mosquito oviposition preferences (Zhang et al. 2020).

Within the past ten years, research has routinely evaluated *B. bassiana* and other entomopathogenic fungi toxicity in adult mosquitoes and found high rates of mortality using different infection protocols (Darbro et al. 2012, Jaber et al. 2016, Buckner et al. 2017, Lee et al. 2019, de Luna-Santillana et al. 2020, Shoukat et al. 2020, Vivekanandhan et al. 2020). One experiment sprayed a 1 × 10^8^ conidia/ml suspension of various isolates of *B. bassiana* into a cage with *Ae. albopictus* and found that multiple isolates had 100% adult mortality at 10 d, with an LT_50_ as low as 4.5 d for the most pathogenic strain (Lee et al. 2019). A different study used a concentration of 3 × 10^8^ conidia/ml and found a mortality rate of 87.5% at seven d with an LC_50_ of 3 × 10^6^ conidia/ml (Shoukat et al. 2020). The concentration of conidia transferred to an ovipositing mosquito is lower than the concentration examined in these studies, which is why our methods used a higher concentration of 1 × 10^9^ conidia/ml. Future research can investigate attractive measures in low-cost oviposition traps to increase the likelihood of ovipositing and the number of eggs laid in the ovitrap, further increasing mycopesticide contact time and removing more vectors from the environment.

Using current trapping methods, many experiments have been successful in transferring conidia from traps to mosquitoes, resulting in mosquito mortality (Scholte et al. 2005, Farenhorst et al. 2008, Howard et al. 2010, Snetselaar et al. 2014, Buckner et al. 2017, Silva et al. 2018). These studies reported that mosquitoes are more attracted to dark colors, yeast-infused water, and synthetic lures than fungi (Snetselaar et al. 2014, Buckner et al. 2017, Paula et al. 2018, Silva et al. 2018). The fungus delivery system has varied widely in trials; passive methods included applying conidia to resting container walls and netting, and active methods in laboratory trials included applying conidia topically, injecting conidia into the thorax, and spraying conidia directly onto adults (Farenhorst et al. 2008, Howard et al. 2010, Mnyone et al. 2010, Johnson et al. 2019, Lee et al. 2019, Ramirez et al. 2019, Shoukat et al. 2020). Our study applied the mycopesticide formula to the substrate with a paintbrush for an even coating that dried on the wooden substrate. Two studies found no repellent effect between nets treated with the fungus and control nets by observing at the number of mosquitoes that traveled toward an odor cue through placed holes in fungus-impregnated netting (Howard et al. 2010, Mnyone et al. 2010). Conversely, George et al. (2013) performed an olfactory choice experiment in which fungus was placed at one end of the Y-tube and found that fungus was an attractant to *Anopheles stephensi* Liston. Using ovitraps and different formulation of *B. bassiana*, we demonstrated *Ae. albopictus* did not exhibit a similar response. The inconclusive body of literature necessitates further research on fungus-induced behavior modifications as well as different strains, formulations, and species of mosquitoes.

*Aedes albopictus* exhibits an ovipositional behavior known as “skip oviposition”, where one gravid female lays eggs from the same gonotrophic cycle in multiple larval habitats (Trexler et al. 1998, Reinbold-Wasson and Reiskind 2021). Thus, multiple eggs from the same gravid female may have been laid at multiple paddles in the same quad-ovitrap. If this occurred with the highest concentration of fungus habitat being selected against, or had a repulsive effect on the gravid mosquito, it would further support our observation that *Ae. albopictus* altered the number of eggs laid due to the presence of fungus. The two highest concentrations of fungus, 1 × 10^9^ and 1 × 10^7^ conidia/ml, were associated with lower medians and numbers of paddles with eggs than the control and 1 × 10^5^ conidia/ml concentration, as would be predicted if there was a repellent effect, causing the gravid female to oviposit at a perceived better location without a high concentration of fungus and then move on to a different site. This may have been occurring during the experiment, as there was a higher likelihood of the presence of eggs on control paddles, indicating that control paddles and paddles with lower concentrations of *B. bassiana* were considered more attractive oviposition sites. Additionally, skip oviposition may also increase the likelihood of fungal transference to the mosquito, as increased contact time results in higher infectivity (de Paula et al. 2008).

One challenge in creating an effective fungus delivery system is the low tolerance of entomopathogenic fungi to sunlight. As fungi degrade quickly in UV light, the traps were placed in shaded and humid areas, which are also mosquito-favored habitats (Fargues et al. 1996, Zimmermann 2007, Dickens et al. 2018). Observations of the postcollection paddles showed sporulated fungal bodies, indicating successful application of the fungus. The current study shows that UV degradation can be minimized with attention to trap placement, aided by temperatures that allow for fungus survival.

## Conclusions

This study was conducted to ascertain the integration feasibility of two existing vector management strategies, entomopathogenic fungi and ovitraps. The results showed larvicidal activity of *B. bassiana* at concentrations at or higher than 1 × 10^6^ conidia/ml against second- and third-instar *Ae. albopictus*. We also demonstrated that gravid *Ae. albopictus* laid fewer eggs on wooden paddles with higher concentrations of *B. bassiana* and the likelihood of egg laying decreased with increasing fungal concentrations. By using a precise concentration of entomopathogenic fungi, infectivity and pathogenicity can be optimized for control measures without sacrificing the number of mosquitoes infected. The use of *B. bassiana* can be integrated with existing integrated vector management strategies to better control vectors and to minimize the spread of insecticide resistance in mosquito populations.

## Supplementary Material

tjac084_suppl_Supplementary_Figure_1
