# Supplementary figures and images for: Larval Mortality and Ovipositional Preference in Aedes albopictus (Diptera: Culicidae) Induced by the Entomopathogenic Fungus Beauveria bassiana (Hypocreales: Cordycipitaceae)
Source: J Med Entomol. 2022 Jul 7;59(5):1687–93. doi: 10.1093/jme/tjac084 (PMC9473649; doi:10.1093/jme/tjac084)

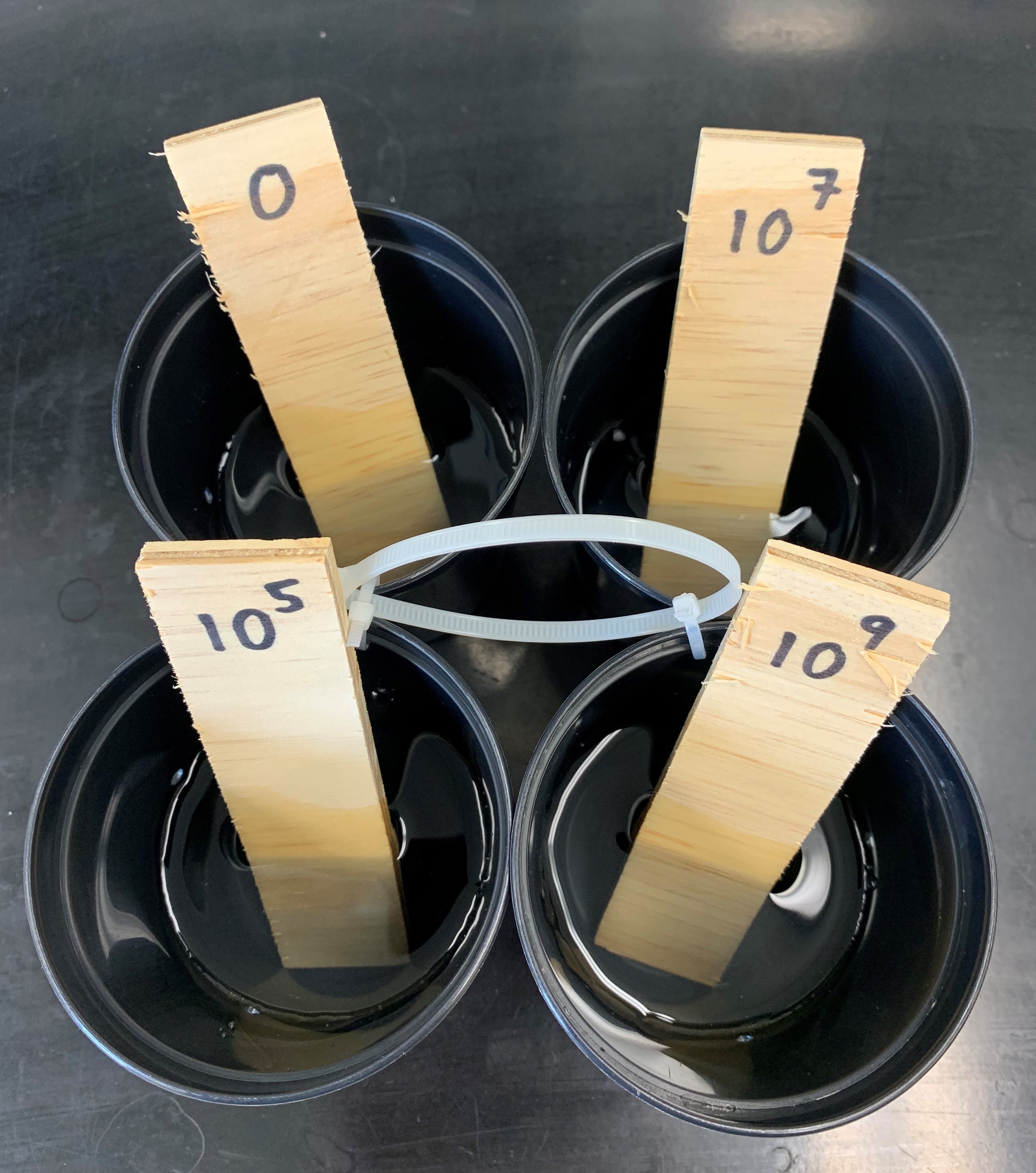

Supplement: tjac084_suppl_Supplementary_Figure_1 [file tjac084_suppl_supplementary_figure_1.jpeg]
